# Food Habit Associated Mycobiota Composition and Their Impact on Human Health

**DOI:** 10.3389/fnut.2021.773577

**Published:** 2021-11-22

**Authors:** Jata Shankar

**Affiliations:** Genomics Laboratory, Department of Biotechnology and Bioinformatics, Jaypee University of Information Technology, Solan, India

**Keywords:** mycotoxin, phytoestrogens, alcohol intake, gut mycobiome, diet habit, COVID-19, mucormycosis

## Abstract

Mycobiota is not only associated with healthy homeostasis in the human gut but also helps to adapt to the environment. Food habits, alcohol consumption, intake of probiotics, and contaminated food with a mycotoxin, often lead to the alteration in the mycobiota composition. Impaired immunity of the host may affect fungal symbiosis leading to mycosis. The human gut adapts to the commensalism fungi belonging to the phylum Ascomycota and Basidiomycota. Diet habits such as plant-or animal-based, phytoestrogens enriched plant products, fat-rich diets also influence the colonization of certain fungal species in the mammalian gut. Food habits or mycotoxin-contaminated food or fungal peptides have an impact on bacterial-fungal interaction and human health. The mycobiota population such as *Fusarium, Humicola, Aspergillus*, and *Candida* are altered due to alcohol intake in alcoholic liver disease. The role of associated gut mycobiota due to irregular bowel habits or lifestyle change has been observed in inflammatory bowel disease. In this review, it has been observed that *Saccharomyces, Aspergillus, Fusarium, Cladosporium, Candida*, and *Malassezia* were the common genus in the human mycobiota. Therefore, this study focused on how diet habits and alcohol intake, among others., influence mycobiota composition that may affect the human immune system or overall health.

## Introduction

The human system harbors many diverse and unculturable species such as bacteria, protozoan, viruses, and fungi, to name a few, which constitute microbiota (1, 2). These species live on and inside the human body, but in comparison with the bacteria, the diversity and abundance of fungi are relatively lower (3). Thus, fungal microbiota or are often less explored (4, 5). The association of the fungal community to human health is a well-known fact (6). The relationships between organisms within a microbial community (i.e., symbiosis) or the unbalance of microbial community composition (i.e., dysbiosis) and their role within a host is an active area of research. Dysbiosis not only permits or promotes the growth of certain fungal species, but is also associated with alteration in internal homeostasis, and influences the systemic immunity of the individuals (7). The alteration of mycobiota composition may depend on types of food intake or due to the recent rise in immune-compromised patients. In addition, fungal communities may also be beneficial in micronutrient extraction, as well as in the production of enzymes and vitamins, to aid digestion (3). Thus, understanding the relationship between host-fungi and food-fungi seems critical for good health.

**Figure 1:**
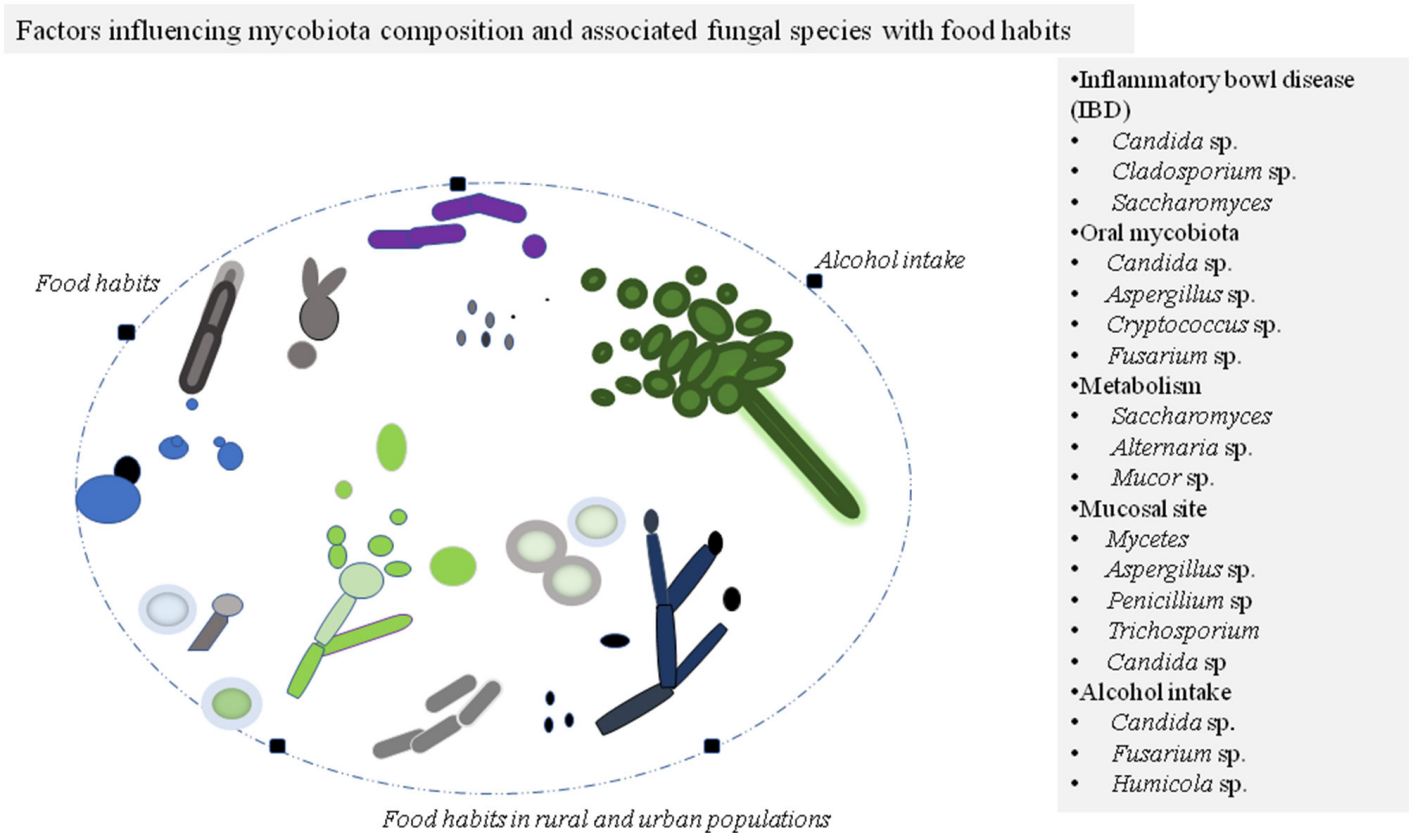
Factors affecting mycobiota composition of the individuals commonly belong to phylum Ascomycota and Basidiomycota. The different morphotypes of fungal species are illustrated in colors for presentation purposes. The modulated fungal species during the disease state and other conditions are listed in the panel.

Recently, there has been a focus on characterizing bacteria in the healthy individual and with disease conditions, with a limited effort on fungal microbiota due to their lower abundance and culturing complications (8). Studies on human microbiota have revealed that predominant mycobiota in the human system belongs to Ascomycota and Basidiomycota phyla with commonly observed genus from *Saccharomyces, Aspergillus, Fusarium, Cladosporium, Candida*, and *Malassezia*, among others (8, 9). Based on the recent development, this mini-review addresses the symbiosis or dysbiosis of human mycobiota associated with food habits or other factors (Figure 1) and their impact on human health.

### Food Habits

Dietary intake/choice of food is an important factor that determines microbial composition in the human gut (10). Maintaining the balance among microbes including fungal species in the gut is critical for better gastrointestinal health (11). The human mycobiota diversity is influenced by the diet of an individual, including fermented food products, bread, as well as alcoholic beverages (12). Yeast cells are the most common microorganism for fermented food products, e.g., cheese (13), bread (14), beer (15), and others (16). Other species such as *Debaryomyces hansenii*, a yeast species, have been detected in salt fermented products and *Penicillium roqueforti* in blue cheese (17). Studies on the decreased intake of bread and beer have been shown to minimize the amount of *Saccharomyces cerevisiae* in human stools (18). In a controlled study, it was examined that *Penicillium* was correlated with a plant-based diet and *Candida* was enriched in animal-based diet participants (12). To decipher the relation between obesity with diets, the variation in the human gut microbiome in obese was assessed and compared with that of non-obese subjects by ITS-based sequencing (19, 20). The fungal species found in non-obese were *Pichia, Candida, Aspergillus, Mucor*, and *Saccharomyces, S. cerevisiae* being the most abundant. No specific changes in fungal species were observed in obese patients, except the absence of a minor phylum *Zygomycota*. In obese patients, *Candida* and *Penicillium* were abundant, while *Mucor* was lacking. In non-obese patients, the Mucor species suggested being associated with weight loss (19). In other studies in obese subjects, the risk of developing cardiovascular diseases and type 2 diabetes was reported (8). The fat-rich diet has affected mycobiota composition in the gut (e.g., the low abundance of *S. cerevisiae*) and possibly led to a shift in microbiota composition in mice (20). Furthermore, this has been found in correlation with a low abundance of *S. cerevisiae* in obese subjects in comparison with the gut of control subjects (19).

To understand the impact of dietary intake on the interaction among fungal and bacterial species, fecal samples from Indian and Japanese adults were subjected for sequencing (21). Indian population having plant-rich polysaccharides in their diet showed the abundance of *Prevotella* and *Candida* in comparison with the Japanese population. Therefore, vegetarian or animal-based diet or both pertaining to good health or diseased-state concerning mycobiota needs more research work (22, 23). In particular, fungal species producing toxins, such as aflatoxin, ochratoxin P, zearalenone, patulin, etc. may be carcinogenic or severely harmful to the host (24, 25). These toxins, either from consumed foods or from the resident mycobiota may lead to adverse effects including their impact on host immune response (26, 27). Thus, the mycotoxin produced from the gut mycobiota also needs investigation. These findings presented here provided a general understanding of how diets (plant- or animal-based, fat-rich, or fermented product) might influence the dynamics of gut mycobiota of the host. Thus, consumption of a balanced diet that could manifest the suitable microbial composition in the gut likely to promote good health.

Several veggies, legumes, grains, especially in soy, are structurally and/or functionally similar to mammalian estrogens, called phytoestrogen (28). It can interfere with steroid biosynthesis (29), thus modulating the free circulating endogenous hormones including estrogens. Stimulatory or inhibitory effects on the growth of the fungal communities have been observed by estrogen/17-β-estradiol (30). For instance, it has been reported the stimulation of the growth of *Candida albicans* by 17-β-estradiol (31). Elevated estrogen levels in the host and occurrence of Candidiasis are often associated (32). The stimulatory effect of 17-β-estradiol allows *C. albicans* to colonize on mucosal surfaces, including the reproductive tract (33). Overgrowth of *C. albicans* in the mucosal surfaces leads to Vulvovaginal candidiasis, characterized by scratching and inflammation (34). On the other hand, 17-β-estradiol showed an inhibitory effect on *Paracoccidioides*, a thermoregulated dimorphic fungus causing systemic mycosis (paracoccidioidomycosis) in Latin America (35). It has been observed that females are about 13–70 times less likely than males to develop the clinical disease and women are resistant to paracoccidioidomycosis (36). Furthermore, 17-β-estradiol has been shown to block/delay the morphogenesis of *Paracoccidioides via* stress responses (Heat shock proteins), signaling pathways through kinase nodes (37). Thus, endogenous hormone or dietary intake of phytoestrogen may have a profound effect on a few fungal species, and during the disease state, the estrogen level may need manipulation *via* restricted diet or supplements.

### Alcohol Intake

Alcohol is often consumed along with food around the world. Owing to chronic alcohol intake, the composition of bacterial and fungal species is altered. Alcoholic liver disease (ALD) is responsible for half of all cirrhosis deaths. Chronic ethanol intake (up to 8 weeks) increased the fungal richness and diversity among mice. The mycobiota population increased significantly in particular such as *Fusarium, Humicola, Aspergillus*, and *Candida*. ALD raised the plasma level of β-glucan in mice (38). It increases the translocation of microbial products of fungal species from the intestinal lumen to the systemic circulation that causes inflammation of the liver. This leads to hepatocyte damage and facilitates the development of ethanol-induced liver disease. However, anti-fungal treatment to mice suppressed fungal overgrowth but also affected liver disease. The distribution of mycobiota was seen in patients with alcoholic hepatitis and compared with that of patients with alcohol use disorder (39). *C. albicans* was primarily observed in patients with alcoholic hepatitis, while normal patients were tested for *Penicillium*. Moreover, anti-*S. cerevisiae* antibodies in serum (ASCA) were measured to determine the systemic immune response to fungal products or fungi. Patients with alcoholic hepatitis had higher levels of ASCA relative to patients with alcohol consumption disorder and non-alcoholic patients. ASCA were also observed in other subjects such as IBD (40, 41) celiac diseases (42), thus antibodies against *S. cerevisiae* in serum could act as a marker for the inflammatory response (43). Additionally, the fungi-bacteria association has been observed to correlate positively in alcoholic hepatitis patients (44). Thus, screening of fungal species or antibodies against them could be recommended in these subjects to assess the health risk.

### Food Habits in Rural and Urban Populations

The fungal composition of our gut is largely affected by the consumption of packed food such as dairy products, meat products, and frozen vegetables in urban environments vs. fresh food products in local rural environments (45). To understand the influence of the environment on fungal composition in humans, a cohort of 151 Amerindians living in remote communities (French, Guinea) was studied at 4-year intervals. They showed a rich diversity of fungi relative to people living in western cultures. People living in industrial areas have *C. albicans* as a predominant species in their intestines (11). Furthermore, *Candida krusei* and *S. cerevisiae* were found to be abundant in remote communities. *C. albicans* were more common in females and crowded areas. *C. krusei* and *S. cerevisiae* were known to be associated with foodborne by plants and water. It was proposed that there may be cross-transmission of the strains of *C. albicans* between humans and animals (11). A wide diversity of fungi has been found in the human gastrointestinal (GI) tract and detected in stool samples. Fungi found in stools are highly affected by food or the oral cavity. *S. cerevisiae*-free diet renders this strain undetectable in stools. The level of *C. albicans* in the stool has correlated with the cleaning of teeth (18). The study of Sun et al. (46) also studied the fecal mycobiome using metagenomic sequencing to associate fungal communities in the rural and urban populations of China. In brief, *S. cerevisiae* was more abundant while *Candida dubliniensis* was low in abundance in urban compared with rural population suggesting that distinct food habits in the shaping of gut mycobiota composition. The enrichment of fungal species in the gut of these populations may modulate the metabolism of the host to confer good health need more such studies.

## Impact of Mycobiota Composition on Human Health

The mycobiota is a part of human microbiota and plays important role in regulating innate and adaptive immune homeostasis (47–49). While the mycobiota composition of the host may have an independent effect on the microbial environment of the intestine and immune development (50), the balance of microbial communities in providing immunity to protect from invading pathogens to the immune dysregulation is critical for health benefits to human (47, 51). The dynamics of fungal species, such as *Malassezia, Candida, Aspergillus, Cladosporium, Saccharomyces*, and *Penicillium*, among others, are reported in various health conditions (Table 1). Therefore, mycobiota associated with gastrointestinal diseases were discussed here.

**Table 1:** Impact on human health due to the alteration in mycobiota composition.

**Food habits**	**Abundance of fungal species**	**References**
Salt fermented food products	*Debaryomyces hansenii*	(17)
Blue cheese	*Penicillium roqueforti*	(17)
Bread and beer	*S. cerevisiae*	(18)
Plant-based diet	*Penicillium*	(12)
Animal-based diet	*Candida*	(12)
Plant rich polysaccharides	*Prevotella* and *Candida*	(21)
**The potential impact on human health**	**Mycobiota composition**	
Obese patients	Abundance of *Candida* and *Penicillium* and low abundance of *Mucor*	(20)
Candidiasis	Abundance of *Candida*- elevated estrogen levels	(32)
Alcoholic liver disease (ALD)	Increased fungal load—*Candida, Fusarium, Humicola, Aspergillus*	(38, 39)
Mucosal site infections	Presence of mycetes, *Aspergillus, Penicillium*, and *Trichosporon* sp.	(3, 34, 52–54)
	Increased *Candida sp*.	
Inflammatory bowel disease (IBD): Crohn's disease (CD) and ulcerative colitis (UC)	Increased levels of *Candida*, reduced levels of *Cladosporium* sp.	(55–57)
	Decline in *Saccharomyce*s *cerevisiae*	
Oral and lung infection	Overgrowth of *Candida, Aspergillus, Cryptococcus*, and *Fusarium*	(58–60)
Asthma	Increase in *Alternaria* and *Aspergillus* sp.	(61–63)
Allergic fungal rhinosinusitis (AFRS)	Sensitization to *Aspergillus, Bipolaris, Curvularia, Alternaria*, and *Fusarium*	(64, 65)
Allergic bronchopulmonary Aspergillosis (ABPA)	Increased IgE concentration against *Aspergillus* and *Penicillium*	(66)

### Gut Mycobiota and Associated Diseases

The chronic and excessive intestinal inflammation due to the resident or foreign antigens causes inflammatory bowel disease (IBD) (67). It is categorized into two main clinical conditions, namely Crohn's disease (CD) and ulcerative colitis (UC), which are due to the activation of the immune response against certain microbiota in the gut influenced by environmental factors (7, 68). It is characterized by inflammation of the gastrointestinal tract, altered bowel habits, and abdominal pain. The fungal diversity of the gastrointestinal tract is contributed by many species, including *Penicillium, Candida, Aspergillus, Saccharomyces, Cryptococcus*, and *Malassezia* (57). In pediatric subjects, stool samples were collected from IBD patients and healthy subjects and were characterized by deep sequencing of rRNA gene segments specific to the fungal domain. *Pichia jidanii, Candida* species, and *Torula* yeast were abundant in IBD patients. The healthy subjects showed *Clostridium cladosporioides* dominance (56). In IBD patients, particularly CD was screened with antibodies against *S. cerevisiae*, suggesting an inappropriate immune response to this fungus (57). In addition, the study of Jain et al. (69) showed the colonization of *D. hansenii*, a yeast species, at intestinal wounds of mice and inflamed mucosal tissue of CD human subjects, and mucosal healing dysfunction. Thus, inflammation in the gut either because of yeast colonization and/or immune suppressive therapy needs more studies. Genetic variation such as polymorphism in CLEC7A, a gene encoding for Dectin-1, can induce UC in mammals due to a weak immune response against fungi (55). Dectin-1, a pattern recognition receptor for β-glucan, is involved in adaptive immune response *via* T-helper cells, and the polymorphism in the receptor makes the host susceptible to invasive infections (70). The work of Tang et al. (71) used Dectin-1 deficient mice to demonstrate that Dectin-1 regulates regulatory T (Treg) cell differentiation under the influence of microbiota composition, implying the role of Dectin-1 in intestinal immunity. Chitooligosaccharide (COS), a derivative of chitosan, possesses antimicrobial activity. The alteration in the diversity of mycobiota was observed in colorectal cancer (CRC) patients before and after COS induction. In addition, it has been associated with decreased symptoms of colitis-induced CRC (72). The data showed that the abundance of two phyla in intestinal mycobiota: Ascomycota and Basidiomycota (73).

Fungal species such as *Candida* and *Aspergillus* release mycotoxins into non-neuronal tissues that pass through the bloodstream. These toxins target astrocytes and oligodendrocytes, as a result of which the blood-brain barrier is weakened followed by degradation of myelin (74). The above studies showed mycobiota plays a critical in modulating the metabolism of host and intestinal immunity. It was suggestive that the intestinal environment could be managed by modulating the mycobiota composition to achieve a healthy gut environment.

## Mycobiota and Impact on Host Immune System

Fungi are also part of oral microbial communities along with bacterial species and these microbes enter to human gut through ingested foodstuff (75). The fungal mycobiome has been explored in the oral cavity from healthy individuals and reported a total of 101 species, 74 cultivable, and 11 non-cultivable (58). *Candida* was followed by *Cladosporium, Aurobasidium, Saccharomyces, Aspergillus, Fusarium*, and *Cryptococcus*, among the most common species. Four of these species, namely *Candida, Aspergillus, Fusarium*, and *Cryptococcus*, are pathogenic to immunocompromised humans when present in abundance (76–79). In the oral microenvironment, *C. albicans* associates with *Streptococcus* species to promote bacterial colonization (80), and synergistic interaction between fungal-bacterial species contribute to the development of multi-microbial biofilm (81–83). Thus, interspecies interaction and microbial biofilm formation in the oral cavity surfaces need more such studies, consequently, enable better strategies to treat oral diseases. In rural areas or unhygienic environments, human beings are often exposed to fungal spores that disturb the mycobiota composition under healthy conditions (84). In adverse conditions, inhaled fungal spores cause severe health deterioration (7). In another study, the mouth and lung fungal microbiota was compared between healthy individuals and recipients of lung transplants (60). *Candida* species were found in the oral wash of lung transplant recipients because of antibiotic therapy and immunosuppressant. There was limited fungal ITS amplification in the bronchoalveolar lavage of healthy individuals, whereas detectable fungi of *Candida, Aspergillus*, or *Cryptococcus* were present in lung transplant recipients (59). Thus, balanced oral microbial communities are required to be a healthy oral system. If microorganisms are altered may enhance the risk of oral diseases.

The human immunity system is affected by microbiota composition, and secondary metabolites and/or peptides produced by them (85, 86). To understand the role of the fungal component in modulating immunity, when the Influenza A virus was infected in antibiotic-treated mice, they were more vulnerable to colitis-induced dextran sodium sulfate (DSS) and displayed decreased CD8^+^ T cells. The protective immunity resumes adequately when commensal fungal species, such as *S. cerevisiae* or *C. albicans* was administered. It was concluded that the immune response may have been produced by mannan, an abundant component of the fungal cell wall. It was therefore noted that the function of the immunocyte was dependent on the composition of the mycobiota in the organism (87). Mucosal and systemic fungal infections cause CD4^+^ T cells to respond to induce Th17 or Th1 cells (47). During the dysbiosis of gut microbiota, the CD4^+^ T cells dysfunction has been observed in childhood atopic subjects (88). *C. albicans* and *Aspergillus fumigatus* are pathogenic fungi and elicit antigen-presenting cells to produce effector T-helper cells against these microorganisms (47, 89–91). On the other hand, *S. cerevisiae*, a non-pathogenic yeast, and *A. fumigatus* were observed inducing both the Th1 and Th17 subsets of CD4^+^ T cells (92, 93). Thus, pathogenic or non-pathogenic fungal spores can promote T-cell response. However, systemic antibodies in humans against major inducers such as *C. albicans* showed protection against disseminated *C. albicans* or *Candida auris* (94). Furthermore, the production of CARD9-dependent antibodies repertoire in shaping host immunity implicated the role of fungal communities in the human gut (94). Therefore, certain mycobiota composition, their interactions, and/or fungal cell wall components in the microbial environment allow individuals to may remain healthy. On the other hand, dysbiosis or the alteration of mycobiota structure and/or function may occur as a consequence of infection, e.g., COVID-19. The study of Zuo et al. (95) observed a significant change in the fecal mycobiota in COVID-19 patients in comparison with control subjects. These patients showed an increased abundance of opportunistic fungal pathogens, such as *C. albicans, C. auris, Aspergillus flavus*, and *Aspergillus niger*, in their fecal samples. Thus, impaired immunity during the infection and delay in recovery of immunity allows opportunistic pathogens to colonize in the host. Additionally, patients recovered from the COVID-19 infection but probably not their immune system since there were still shown infections with black fungi/mucormycosis with high mortality (96). Therefore, microbial symbiosis or balance of bacteria-fungi-virus communities in the host in shaping innate or acquired immunity to the host presents future research opportunities.

## Conclusion and Perspectives

Microbial micro-environment in the human system and its physiological diversity, or the factors that influence its colonization inside the body, is still at a primitive stage. The dynamics of fungal species belonging to *Malassezia, Candida, Aspergillus, Cladosporium, Saccharomyces*, and *Penicillium*, among others, were among the most common genera associated with symbiosis or dysbiosis in humans. Morphological and metabolic changes in these species are essential for survival within hosts and need better understanding. The bacterial predominance in the oral cavity or intestines to achieve optimal health and the role of fungi in gastro-intestinal ecology that fungi influence bacterial activities through various interactions needs thorough investigation. Moreover, exploration of genes encoding proteins produced by fungal species metabolizing certain substrates could result in food recommendations. Furthermore, the identification of coordinated expression of protein from the resident microbes in the gut microenvironment may assist our understanding of the relationship between bacteria-fungi or host-microbe. *In vitro* or *in vivo* experiments should be performed to identify human microbial communities for a normal healthy condition and those associated with the disease. In particular, it is possible to examine the microbiome composition of individuals with neurological disorders to assess their diet and lifestyle, which promote healthy brain activities and actions. Overall, a sound understanding of the biological properties of the mycobiota could increase our quality of life in coordination with diet, lifestyle, and climate.

## Author Contributions

The author confirms being the sole contributor of this work and has approved it for publication.

## Conflict of Interest

The author declares that the research was conducted in the absence of any commercial or financial relationships that could be construed as a potential conflict of interest.

## Publisher's Note

All claims expressed in this article are solely those of the authors and do not necessarily represent those of their affiliated organizations, or those of the publisher, the editors and the reviewers. Any product that may be evaluated in this article, or claim that may be made by its manufacturer, is not guaranteed or endorsed by the publisher.
